# It gets better with age: Resilience, stigma, and mental health among lesbian, gay, bisexual, transgender and queer persons from Poland

**DOI:** 10.3389/fpsyg.2022.958601

**Published:** 2022-09-20

**Authors:** Karolina Koziara, Magdalena E. Mijas, Andrzej Galbarczyk, Jowita Wycisk, Mateusz P. Pliczko, Karolina Krzych-Miłkowska, Bartosz Grabski

**Affiliations:** ^1^Department of Epidemiology and Population Studies, Faculty of Health Sciences, Jagiellonian University Medical College, Krakow, Poland; ^2^Department of Environmental Health, Faculty of Health Sciences, Jagiellonian University Medical College, Krakow, Poland; ^3^Faculty of Psychology and Cognitive Sciences, Adam Mickiewicz University, Poznan, Poland; ^4^Sexology Lab, Department of Psychiatry, Faculty of Medicine, Jagiellonian University Medical College, Krakow, Poland

**Keywords:** protective factors, social stigma, depression, mental health, sexual minority, transgender persons

## Abstract

Lesbian, gay, bisexual, transgender, and queer populations are disproportionately affected by chronic stress associated with stigma which contributes to health adversities including depression. Negative impact of stigma on health can be alleviated by factors such as resilience. Little is known however on how exposure to stigma, protective factors and mental health change with age among gender and sexually diverse persons. Our study aimed at investigating this issue. Our sample consisted of (i) 245 sexually diverse cisgender women, (ii) 175 sexually diverse cisgender men, and (iii) 98 transgender and gender diverse persons. We collected data through a web-based survey. Linear regression models were performed to investigate the interactions of age and each group of participants for resilience, stigma exposure, and mental health indicators (depression and self-esteem). We hypothesized that resilience and mental health indicators will be positively associated with age in all distinguished groups despite the continued exposure to minority stress. The analysis yielded no significant relationships between stigma exposure and age among study participants. However, we observed significant interaction effects of distinguished groups of participants and age in case of self-esteem, depression, and resilience. Self-esteem and resilience were related positively, and depression was negatively associated with age in all study groups. Additionally, we observed that sexually diverse cisgender men demonstrated significantly increased resilience, reduced depression and higher self-esteem compared to other groups. Although the exposure to stigma did not decrease with age, resilience and self-esteem increased, suggesting that LGBTQ persons manage to thrive despite adversities.

## Introduction

### Health inequalities and minority stress in gender and sexually diverse populations

Sexually and gender diverse (GSD) persons are disproportionately affected by mental and physical health inequalities (Cochran et al., 2003; Cochran and Mays, 2007; Conron et al., 2010; Reisner et al., 2016; Valentine and Shipherd, 2018). Lesbian, gay, bisexual, queer, transgender, and non-binary populations are characterized by increased levels of depression, suicidality and substance use (Plöderl and Tremblay, 2015; Newcomb et al., 2020). Growing number of studies also indicate increased prevalence of physical health problems in members of GSD populations including selected cancers (Blondeel et al., 2016) or diabetes (Beach et al., 2018).

Observed health disparities has been linked in the literature with both exposure to gender and sexuality-based harassment and trauma, as well as proximal minority stressors including expectations of rejection and identity concealment (Coulter et al., 2018; Kassing et al., 2021; Pellicane and Ciesla, 2021). Although the minority stress model (Meyer, 2003), which provides a conceptual framework for research on health in GSD populations, also includes protective factors alleviating the negative consequences of stigma and prejudice on health, most studies focus on investigating health risks and predictors of health adversities instead of strengths and coping strategies (Gahagan and Colpitts, 2017). This approach has been criticized as deficit-focused and obscuring research application for health promotion initiatives in GSD communities as it provides limited knowledge on how members of minority populations achieve and sustain their health (Gahagan and Colpitts, 2017).

### Resilience in studies including gender and sexually diverse populations

One of the concepts particularly promising as a conceptual framework for investigating health in GDS populations within strengths-based perspective is resilience (Colpitts and Gahagan, 2016). It has been also recognized as an essential component of minority stress model (Meyer, 2015). Resilience is a complex, multidimensional psychological phenomenon most commonly defined as an individual ability to endure stress and adversities, with potential biological underpinnings related to stress response regulatory mechanisms, immune responses and neural circuitry function (Feder et al., 2019). Factors contributing to resilience include, but are not limited to, effective emotion regulation, optimism, self-efficacy, active coping and social support (Feder et al., 2019). Higher levels of resilience have been linked in research with better mental health and increased quality of life across various age groups and patient populations (Leppin et al., 2014; Zarzaur et al., 2017; Rosenberg et al., 2018; Färber and Rosendahl, 2020). Similar effects have also been observed for physical health outcomes; for instance in a Swedish cohort study low levels of resilience in adolescence predicted increased risk of liver and lung cancer later in life (Kennedy et al., 2017).

Studies sampling GSD individuals also demonstrate associations between resilience and health outcomes (McElroy et al., 2016; Schnarrs et al., 2020) indicating that resilience may buffer the detrimental effect of minority stress (Meyer, 2015). These studies also point at unique sources of resilience in GSD populations including GSD community connectedness (McConnell et al., 2018), being open about GSD identity (Kosciw et al., 2015) and the ability to use the name consistent with affirmed gender in case of transgender and non-binary youth (Tankersley et al., 2021). Resilience level may also change across the life span. It is both possible that it increases with age through more opportunities to develop effective coping, as well as decreases as a result of sensitization to stress and resources depletion (Feder et al., 2019). The evidence on resilience changes across the life span in GSD and general populations is limited and inconclusive both demonstrating significant differences between various age groups (Monin et al., 2017) and suggesting that resilience remains rather stable across various age cohorts (Linnemann et al., 2020). More research, preferably from diverse cultural contexts and conducted in diverse populations, is needed to better understand its age-related dynamics.

### Other protective factors against minority stress and their age-related dynamics

Another construct which is related to autonomic, hormonal and inflammatory responses to stress (O’Donnell et al., 2008; Liu et al., 2014) and which as such may buffer adverse health consequences of minority stress is self-esteem. It refers to subjective appraisal of self-worth and self-acceptance related to various areas of life (Rosenberg, 1965). High self-esteem has been associated with better general adjustment and happiness (Cheng and Furnham, 2004), better self-rated health (Arsandaux et al., 2019; Jafflin et al., 2019) and general well-being (Hajek and König, 2019; Zhang et al., 2020). On the contrary, low self-esteem is linked to increased depression and anxiety (Sowislo and Orth, 2013). People who maintain high level of self-esteem are also less susceptible to suffer from negative events such as socially threatening situations (Hulme et al., 2012) which constitute the core of minority stress. Sexually diverse persons are characterized by lower self-esteem compared to heterosexual individuals (Bridge et al., 2019) and their self-esteem may be predicted by perceived exposure to minority stress (Mijas et al., 2020). Longitudinal analyses indicate that self-esteem increases during the adulthood and peaks between the ages of 60 and 70 years across various genders and birth cohorts (Orth et al., 2018). Again, studies on age-related changes in self-esteem among GSD persons are less available and not conclusive indicating both positive and negative associations between self-esteem and age (Lyons et al., 2013; Lyons, 2015; Mijas et al., 2020).

Particularly little data is available on the variability of self-esteem or resilience associated with age among gender diverse and non-binary individuals. Investigating this variability seems especially interesting in the context of minority stress exposure and its potential cumulative effects with age.

### Current study context and research objectives

Our study aimed at filling described gaps and exploring the associations between age and selected health-related constructs including resilience and mental health indicators such as self-esteem and depression, as well as minority stress exposure in Polish gender and sexually diverse persons. Polish context may be particularly well-suited for this kind of investigations, given the current social and political situation of GSD communities. This includes continued lack of legal protection from hate speech or discrimination based on sexual orientation or gender identity, no legal recognition of marriage equality and no adoption rights for same-sex couples (ILGA-Europe, 2022). Situation may be even more challenging for transgender and non-binary persons who not only face unique stressors associated with access to gender affirmative treatment or legal gender marker change but also face unique minority stressors including gender dysphoria (Lindley and Galupo, 2020). Taken that into consideration we distinguished three groups of participants to analyze the associations between age and health-related constructs within unique contexts of intersecting gender and sexual identities, namely: (i) sexually diverse cisgender women [SDCW], (ii) sexually diverse cisgender men [SDCM], and (iii) transgender and gender diverse persons [TG and GDP]. Secondary aim of this analysis was to compare levels of minority stress and mental health indicators in distinguished groups of participants. We hypothesized that resilience and mental health indicators will be positively associated with age in all distinguished groups despite the continued exposure to minority stress.

## Materials and methods

### Procedure

Our project focused on health determinants among members of Polish LGBTQ community. The web-administered survey was conducted among adult (18 years and older) persons who self-identified as sexually and/or gender diverse individuals. The data had been collected through Qualtrics platform between January and March 2018. The invitations to participate in this study were distributed through social media, emails, and websites of Polish NGOs supporting the LGBTQ community. The survey took approximately 40 min to complete, and no financial incentives were provided. The study was approved by the Research Ethics Committee of the Institute of Psychology at the Jagiellonian University. Since the survey included questions that might trigger potentially distressing emotional responses (i.e., questions on violence and rejection motivated by prejudice and discrimination), participants were offered free consults with team members as well as provided with contact details to local LGBTQ counseling services, at the end of the study.

### Participants

Our sample included 518 sexually and gender diverse persons who participated in the survey. The average age of the study participants was 26.92 (*SD* = 8.53, *mdn* = 24) with the minimum of 19 years and the maximum of 70 years of age. Participants who indicated gender identity other than their assigned gender were allocated to transgender and gender diverse persons group [TG and GDP]. Participants who indicated gender identity consistent with their assigned gender were split into two groups based on their gender: sexually diverse cisgender men [SDCM] and sexually diverse cisgender women [SDCW]. Due to limited number of gender diverse participants in our sample the same was not possible in the case of TG and GDP group. The sample consisted of 245 SDCW, 175 SDCM, and 98 TG and GDP.

### Measures

The survey comprised sociodemographic questions, including gender assigned at birth (*male*; *female*; *I don’t want to answer this question*), multiple choice question concerning gender (*a woman; a transgender woman; a woman with transgender past; a man; a transgender man; a man with transgender past; a transgender person; a transsexual person; a queer person; a non-binary person; an intersex person; other, please specify*) and sexual identities (*lesbian woman; gay man; bisexual person; heterosexual person; asexual person; pansexual person; queer person; I don’t label my sexual identity; other, please specify*), year of birth, the size of the place of residence (*village, city < 10°K inhabitants; city of 10°K to 100°K inhabitants; city of 100°K to 500°K inhabitants; city of 500°K to 1°M inhabitants; city > 1°M inhabitants*), and whether monthly income is enough to cover all necessary expenses (*yes, easily; yes, with some difficulty; yes, with great difficulty; no, it’s not enough; I don’t want to answer this question*). We also used questionnaires to assess self-esteem, resilience, depression, and exposure to sexual minority stigma.

To evaluate the level of self-esteem in the study sample The Rosenberg Self-Esteem Scale (RSES) was used (Rosenberg, 1965). RSES consists of 10 items describing feelings of self-worth which are rated on four-point Likert-type scale (ranging from 1 “strongly agree” to 4 “strongly disagree”). Polish adaptation of this questionnaire is characterized by good psychometric properties (Łaguna et al., 2007). Higher scores indicate higher self-esteem.

To capture the individual ability to cope with various stressors The Resilience Measurement Scale SPP-25 (Ogińska-Bulik and Juczyński, 2008) was used. This Polish questionnaire consists of 25 items, rated on five-point Likert-type scale. It consists of five factors including: (1) perseverance and self-determination, (2) openness to new experiences and sense of humor, (3) personal competence to cope and tolerance of negative emotions, (4) tolerance of failure and treating life as a challenge, (5) optimistic attitude toward life and ability to mobilize oneself in difficult situations. The scale is characterized by satisfactory internal validity and test-retest reliability (Ogińska-Bulik and Juczyński, 2008). The higher average score, the greater the level of individual resilience.

We also used The Daily Heterosexist Experiences Questionnaire (DHEQ) (Balsam et al., 2013) measuring the perceived exposure to heterosexism among LGBTQ persons. The questionnaire consists of 50 items which participant’s rate using a six-point scale in terms of the degree to which these experiences were stressful to them. The response format was as follows: 0 = “Did not happen/not applicable to me,” 1 = “It happened, and it bothered me not at all,” 2 = “It happened, and it bothered me a little bit,” 3 = “It happened, and it bothered me moderately,” 4 = “It happened, and it bothered me quite a bit,” and 5 = “It happened, and it bothered me extremely.” The questionnaire consists of nine factors, of which the following six were included in the study: Harassment—a factor capturing verbal abuse, and discrimination based on sexual identity; Victimization—a factor describing exposure to physical abuse based on a non-heterosexual identity; Vigilance—a factor capturing the effort made to conceal one’s own sexual or gender identity; Isolation—a factor capturing feelings of loneliness experienced as a result of being a LGBTQ person; Vicarious trauma—stress resulting from learning about discrimination and abuse experienced by other LGBTQ people and Family of origin—a factor illustrating experiences of rejection by the family of origin. The overall psychometric quality of Polish adaptation of this questionnaire was satisfactory (Mijas and Koziara, 2020).

The study also included The Center for Epidemiologic Studies Depression Scale–Revised (CESD-R) (Eaton et al., 2004). This scale consists of 20 items, covering the most common depression symptoms, rated on five-point Likert-type scale (ranging from 0 “not at all or less than one day” to 4 “nearly every day for the last 2 weeks”). Higher scores indicated greater level of depression. Polish adaptation of CESD-R questionnaire was characterized by good psychometric properties (Koziara, 2016).

### Statistical analysis

Statistical analysis was conducted using *R Studio* with *ggplot2*, *tidyverse*, *rstatix*, and *jtools* packages. First, we compared three distinguished groups of the participants (SDCW, SDCM, and TG and GDP) across demographic variables through a series of logistic regressions with age as a covariate. Next, the analysis of covariance was performed to compare self-esteem, resilience, depression, and perceived exposure to stigma in distinguished groups. Given the significant age difference between participants from all three groups, the analyses were adjusted for grand mean centered age. Finally, we utilized the linear regression to assess the relationship between age and resilience, exposure to stigma, self-esteem and depression in all distinguished groups of participants.

## Results

### Preliminary analyses

The preliminary analyses yielded significant age differences between distinguished groups of participants [*F*(2, 515) = 25.40, *p* < 0.001]. SDCM were significantly older than SDCW (*p* < 0.001) and significantly older than TG and GDP (*p* < 0.001). There was no significant difference in age between groups of SDCW and TG and GDP (*p* = 0.464). Socio-demographic details are presented in Table 1. Logistic regressions indicated that persons assigned female at birth were more than twice more likely to be TG and GDP compared to individuals assigned male at birth (*B* = 0.74, *p* = 0.004, *OR* = 2.09, *CI* = 1.29, 3.47). Additionally, we observed in a logistic regression model a significant difference related to financial difficulties experienced by study participants (sufficient vs. not sufficient income to cover the basic needs)–both SDCW (*OR* = 0.44, *CI* = −1.36, −0.29) and TG and GD persons (*OR* = 0.31, *CI* = −1.80, −0.56) more often declared receiving wage under their basic needs as compared to SDCM. Both SDCW and TG and GDP also more often compared to SDCM refused to answer the question concerning their income (*OR* = 3.03, 95%*CI*: 0.42, 1.89 and *OR* = 3.71, *CI* = 0.51, 2.16, respectively). Correlation matrix and Cronbach’s alpha for studied variables are presented in Table 2.

**TABLE 1:** Comparison of sociodemographic characteristics across distinguished groups of participants.

Variable	SDCW (*n* = 245)		SDCM (*n* = 175)		TG&GDP (*n* = 98)	
			
	*M (SD)*	β *(SE)*	*M (SD)*	β *(SE)*	*M (SD)*	β *(SE)*
Age	25.45 (7.37)	1.15 (0.97)	30.45 (9.55)	6.15 (1.03)***	24.30 (7.28)	Ref
		−5.00 (0.81)***		Ref		−6.15 (1.03)***
	%	*OR* [95% *CI*]	%	*OR* [95% *CI*]	%	*OR* [95% *CI*]
Place of residence (>500 k)	48	0.99 [0.62, 1.59]	57	1.45 [0.88, 2.38]	48	Ref
		0.69 [0.46, 1.01]		Ref		0.69 [0.42, 1.14]
Income (sufficient)	89	1.63 [0.76, 3.38]	93	2.47 [1.07, 5.78]*	84	Ref
		0.66 [0.31, 1.35]		Ref		0.40 [0.17, 0.94]*
Education (university)	67	1.31 [0.81, 2.13]	72	1.70 [1.01, 2.87]*	60	Ref
		0.77 [0.50, 1.18]		Ref		0.59 [0.35, 0.99]*

SDCW, Sexually Diverse Cisgender Women; SDCM, Sexually Diverse Cisgender Men; TG&GDP, Transgender and Gender Diverse Persons; *p < 0.05; ***p < 0.001.

**TABLE 2:** Correlation matrix and descriptive statistics for studied variables.

Variable	Resilience	Stigma exposure	Self-esteem	Depression	Cronbach’s α	Skewness	Kurtosis
Resilience	−	−0.23***	0.68***	−0.59***	0.94	–0.55	0.33
Stigma exposure	−	−	−0.27***	0.31***	0.82	0.71	1.08
Self-esteem	−	−	−	−0.75***	0.92	–0.12	–0.62
Depression	−	−	−	−	0.96	0.51	–0.84
Age	0.30***	−0.14***	0.41***	−0.41***	−	1.10	2.90

***p < 0.001.

### Stigma and health indicators among distinguished groups

**FIGURE 1:**
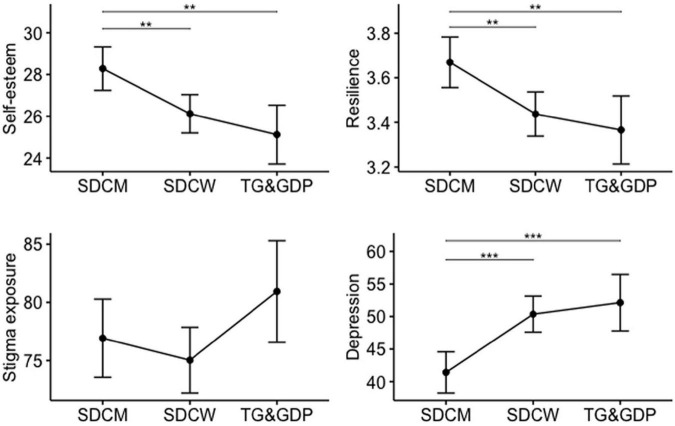
Comparison of self-esteem, resilience, depression, and stigma exposure across separate groups of participants (adjusted for age). SDCW, Sexually Diverse Cisgender Women; SDCM, Sexually Diverse Cisgender Men; TG and GDP, Transgender and Gender Diverse Persons; **p < 0.01, ***p < 0.001.

The comparison of self-esteem (SES), resilience (SPP-25), exposure to stigma (DHEQ), and depression level (CESD-R) across distinguished groups of participants revealed several significant differences (Table 3). SDCM were characterized by significantly higher resilience compared to SDCW and TG and GDP. Similar pattern was observed in the case of self-esteem i.e., SDCM showed significantly higher self-esteem compared to SDCW and TG and GDP. We observed no significant differences in self-esteem and resilience between SDCW and TG and GDP, however in both cases SDCW scored higher. TG and GDP were characterized by significantly increased exposure to stigma associated with being an LGBTQ person compared to SDCW. We also observed significantly higher level of depression in both SDCW, and TG and GDP compared to SDCM (Figure 1 and Table 3).

**TABLE 3:** Results of analysis of covariance for resilience, stigma exposure, self-esteem, and depression.

Variable	*M (SD)*	Ancova results* (*df*)			*p*
Self-esteem	26.70 (6.85)	7.36 (2, 390)			< 0.001

			**Tukey HSD post hoc**		
			
			SDCW (*ems* = 26.1) TG&GDP (*ems* = 25.1) TG&GDP	SDCM (*ems* = 28.3) SDCM SDCW	< 0.001
					< 0.001
					0.198

Resilience	3.51 (0.71)	6.32 (2, 395)			0.002

			**Tukey HSD post hoc**		
			
			SDCW (*ems* = 3.44) TG&GDP (*ems* = 3.37) TG&GDP	SDCM (*ems* = 3.67) SDCM SDCW	< 0.001
					< 0.001
					0.470

Stigma exposure	2.40 (0.67)	2.53 (2, 467)			0.080

			**Tukey HSD post hoc**		
			
			SDCW (*ems* = 2.34) TG&GDP (*ems* = 2.53) TG&GDP	SDCM (*ems* = 2.40) SDCM SDCW	0.990
					0.072
					0.041

Depression	47.49 (20.51)	11.01 (2, 367)			< 0.001

			**Tukey HSD post hoc**		
			
			SDCW (*ems* = 50.4) TGD (*ems* = 52.1) TGD	SDCM (*ems* = 41.4) SDCM SDCW	< 0.001
					< 0.001
					0.484

*Adjusted for age; ems, estimated marginal means (age adjusted).

SDCW, Sexually Diverse Cisgender Women; SDCM, Sexually Diverse Cisgender Men; TG&GDP, Transgender and Gender Diverse Persons.

### Age-related dynamics of stigma exposure, resilience, and other health indicators

**FIGURE 2:**
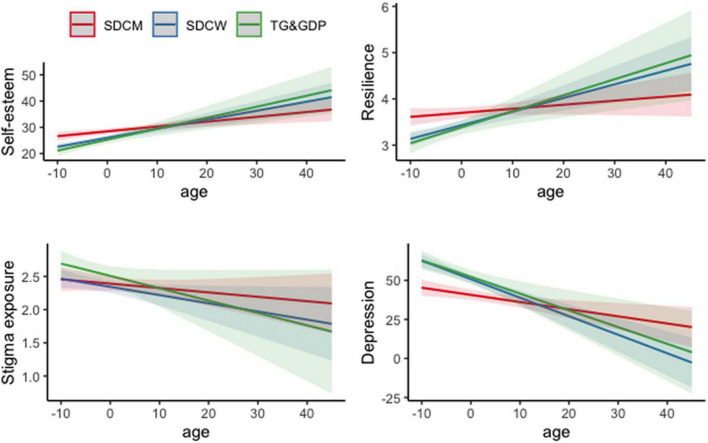
Interaction plot for age (centered) and separate groups of participants across studied variables.

We then further investigated the relationships between age and self-esteem, resilience, exposure to stigma and depression in distinguished groups (Figure 2 and Table 4). Age was associated with increased self-esteem and decreased depression symptoms in all distinguished groups of participants. Models for self-esteem and depression as dependent variables showed satisfying parameters and medium adjusted *R*^2^ values (*R*^2^ = 0.20 and *R*^2^ = 0.23, respectively). Although in case of SDCM we did not observe significant association between age and resilience, both in SDCW and TG and GDP this relationship was significant and positive. The size of the effect of age on resilience in both groups was however small and adjusted *R*^2^ for this model was low (*R*^2^ = 0.12). The model with exposure to stigma as a dependent variable was characterized by the least satisfying parameters and revealed no significant associations between exposure to stigma and age.

**TABLE 4:** Linear models for self-esteem (RSES), resilience (SPP-25), stigma (DHEQ), and depression (CESD-R).

Variable	Estimation *(SE)*	95% *CI*	*t* value	*p*
**Self-esteem**	***F*_(5, 388)_ = 20.59, *p* < 0.001, *R*^2^_adj_. = 0.20**			

SDCM^a^	28.46 (0.55)	27.38, 29.54	51.76	< 0.001
SDCW	−2.46 (0.72)	−3.87, −1.05	–3.43	0.001
TG and GDP	−3.18 (0.92)	−4.99, −1.37	–3.46	0.001
Age^b,c^	0.18 (0.05)	0.08, 0.29	3.53	< 0.001
SDCW x Age^c^	0.16 (0.08)	0.01, 0.32	2.03	0.043
TG and GDP x Age^c^	0.24 (0.11)	0.02, 0.49	2.16	0.031

**Resilience**	***F*_(5, 393)_ = 12.20, *p* < 0.001, *R*^2^_adj_. = 0.12**			

SDCM^a^	3.70 (0.06)	3.58, 3.82	62.06	< 0.001
SDCW	−0.27 (0.08)	−0.42, −0.12	–3.45	0.001
TG and GDP	−0.31 (0.10)	−0.51, −0.12	–3.12	0.002
Age^b,c^	0.01 (0.01)	−0.01, 0.02	1.54	0.124
SDCW x Age^c^	0.02 (0.01)	0.01, 0.04	2.43	0.016
TG and GDP x Age^c^	0.03 (0.01)	0.01, 0.05	2.19	0.029

**Stigma exposure**	***F*_(5, 465)_ = 3.16, *p* = 0.008, *R*^2^_adj_. = 0.02**			

SDCM^a^	2.39 (0.06)	2.83, 2.50	43.41	< 0.001
SDCW	−0.05 (0.07)	−0.19, 0.09	–0.67	0.501
TG and GDP	0.12 (0.09)	−0.06, 0.29	1.27	0.203
Age^b,c^	−0.01 (0.01)	−0.02, 0.01	–1.23	0.221
SDCW x Age^c^	−0.01 (0.01)	−0.02, 0.01	–0.71	0.476
TG and GDP x Age^c^	−0.01 (0.01)	−0.3, 0.01	–1.07	0.287

**Depression**	***F*_(5, 365)_ = 23.18, *p* < 0.001, *R*^2^_adj_. = 0.23**			

SDCM^a^	40.70 (1.66)	37.44, 43.96	24.54	< 0.001
SDCW	10.08 (2.16)	5.83, 14.33	4.66	< 0.001
TG and GDP	11.53 (2.78)	6.05, 17.00	4.14	< 0.001
Age^b,c^	−0.46 (0.15)	−0.76, −0.15	–2.97	0.003
SDCW x Age^c^	−0.73 (0.23)	−1.19, −0.27	–3.12	0.003
TG and GDP x Age^c^	−0.61 (0.32)	−1.25, 0.02	–1.90	0.058

SDCW, Sexually Diverse Cisgender Women; SDCM, Sexually Diverse Cisgender Men; TG and GDP, Transgender and Gender Diverse Persons; ^a^Reference category; ^b^Represents regression slope for SDCM; ^c^Grand mean centered.

## Discussion

The aim of our study was to extend current knowledge on associations between age and selected health indicators in gender and sexually diverse populations. Specifically, we investigated whether the levels of resilience, reported minority stress and mental health indicators such as depression and self-esteem were associated with age in groups of transgender and non-binary persons, sexually diverse cisgender women and sexually diverse cisgender men. Secondary aim of this analysis was to compare the levels of resilience, self-esteem, depression, and exposure to stigma in the distinguished groups of participants.

Although we did not observe significant relationship between exposure to stigma and age, we demonstrated that age was significantly and positively associated with self-esteem and negatively associated with depression in all the distinguished groups of participants. This suggests that although the exposure to various minority stressors does not significantly change with age, mental health indicators improve among GSD persons. The magnitude of this change however varies across the distinguished groups of participants. Observed associations are consistent both with previous studies indicating steady increase in self-esteem with age across gender, nationality, ethnicity, or birth cohort (Orth et al., 2018), as well as research demonstrating negative and significant associations between depression and self-esteem (Orth and Robins, 2013). There is also some evidence in the literature that depression levels drop throughout the adulthood (Mirowsky and Ross, 1992; Hasin et al., 2005). Our analysis indicate that these developmental trajectories can also be observed in populations burdened with constantly increased stress load and persist despite the devastating effect of stress on mental health.

In the case of resilience positive and significant associations with age were observed in sexually diverse cisgender women and gender diverse persons, but not in sexually diverse cisgender men. The effect of age on resilience was the greatest in case of TG and GDP and somewhat smaller in SDCW. These differences can be explained for instance within stress-buffering model (Wheaton, 1983). According to this framework increased stress is usually associated with greater received support which in turn strengthens individuals’ resilience and self-esteem. Given that SDCM were characterized by the highest levels of resilience and self-esteem in our sample, it is possible that they had sufficient coping resources and were not in need of reaching out for support. To put it in other words, those who needed the least support, got the least benefits that come along.

Another explanation of observed differences is the willingness to seek for professional help, and the ability to make the most of the received support. For instance, there are several gender differences related to the efficacy of psychotherapy, with women receiving the greatest benefits from it (Ogrodniczuk, 2006; Békés et al., 2016; Deter et al., 2018). In case of TG and GDP contacting mental health professionals is essential to obtain access to gender affirmative interventions (Koziara et al., 2021). It is possible that TG and GDP are more likely to reach for professional support or receive such support during the diagnostic and transitioning process. Additionally, accomplished social and medical transition which inevitably requires time is related to improved mental health outcomes (Dhejne et al., 2016) and enables further personal development which might have been hindered by unresolved gender dysphoria (Cooper et al., 2020). To sum up, despite the initially higher level of self-esteem and resilience, cisgender men seem to maintain the stable level of both, while cisgender women and gender diverse persons demonstrate significant increase with age.

Despite social change and increasing acceptance toward sexually and gender diverse persons, the exposure to stigma and discrimination continues to be one of the greatest challenges to mental health in gender and sexually diverse populations (Ross et al., 2018; Pachankis et al., 2021). This is particularly true in case of Poland which has been recently again recognized by ILGA-Europe as a country with the worst human rights situation of LGBTQ people in the European Union (ILGA-Europe, 2022). According to the latest report on the social situation of gender and sexually diverse people living in Poland published by the Campaign Against Homophobia (Winiewski and Świder, 2022) the majority (59%) of respondents revealed being exposed to verbal abuse, slightly more than one person out of 10 (14%) reported exposure to physical violence and almost one in five people (22%) experienced sexual violence based on their gender or sexuality. These rates were additionally increased in case of youth for all types of violence including verbal (65%), physical (18%), and sexual (26%) abuse (Winiewski and Świder, 2022). School youth was also characterized by alarmingly high prevalence of suicidal ideations which were reported by nearly three quarters (74%) of participants (Winiewski and Świder, 2022).

Although we did not observe significant associations between exposure to heterosexism and age, decreased coping resources and increased levels of depression in younger participants indicate the urgent need to take protective measures focused on youngest members of the GSD community. Importantly, such interventions should focus not only on individual but also socio-ecological and structural dimensions of stigma which contribute to health inequalities in the first place and decrease the effectiveness of psychological interventions aimed at reducing the impact of stigma on health (Hatzenbuehler, 2016). Tailored initiatives, such as inclusive education or mental health support for GSD youth, introduced at the right moment, help to alleviate the negative outcomes resulting from exposure to social prejudice and exclusion during this critical developmental period (Snapp et al., 2015; Phillippi et al., 2021). Additional comparisons revealed that this may be particularly true for transgender persons and sexually diverse cisgender women who were characterized by significantly reduced levels of protective factors such as self-esteem and individual resilience and increased depression levels compared to sexually diverse cisgender men. Previous studies indicated that the combination of both social and biological determinants contributes to greater burden of mental health issues in cisgender women as compared to cisgender men (Riecher-Rössler, 2017). This gender gap also includes differences in self-esteem and resilience which are most likely attributable to socioeconomic, sociodemographic, and gender-equality factors (e.g., Bleidorn et al., 2016; Lowe et al., 2022).

Consistently, gender differences in depression prevalence constitute one of the most often replicated effects in psychiatry indicating that women tend to have greater risk of depression compared to men (Kessler et al., 2003). According to the meta-analysis conducted by Patten (Patten et al., 2016), in the case of men, the prevalence of depression is also steadily decreasing over time, while in the case of women, the slope is steeper, leading to greater decrease in depression risk. Given that the majority of TG and GDP in our sample was assigned female at birth, the similarities between SDCW and TG and GDP related to depression level and its association with age may to some extent reflect the effects of gendered socialization, burden with structural stressors and gender stereotyping on health.

### Limitations and future directions

Our study had several limitations including small size of the transgender group, which prevented us from conducting additional comparisons, non-probability sampling, and cross-sectional design which limited any conclusions about causality. Future, preferably longitudinal studies, should further investigate the associations between age and such variables as resilience, depression, and self-esteem in GSD populations. They could also explore to greater extent alternative sources of resilience such as positive identity (e.g., Pereira and Silva, 2021) or dispositional mindfulness (Salvati et al., 2019), along with their age-related dynamics. One of the strengths of this analysis is the inclusion of not only cisgender men and women of various sexual identities but also transgender and non-binary persons in the study. This is particularly important given the relative dominance of studies conducted in gay men within the field of LGBTQ health (Salvati and Koc, 2022). We, also, managed to provide data from unique and little recognized in previous studies Central European context and presented effects that may inspire future studies.

In conclusion, our study supported the hypothesis that even if age is not related to the positive change in stigma exposure; it significantly predicts the positive change over time in the case of all health-related constructs included in the analysis. Even in the context of continued exposure to stigma, LGBTQ persons seem to get more resilient, less depressed, and have better self-esteem with age. However, this association is more consistent and stronger in the group of cisgender women, and transgender and gender diverse individuals. This supports the conclusion that professional support provided to LGBTQ persons should be tailored to needs of those who seek help, including their gender, age, and the time point in the courses of their lives.

## Data availability statement

The raw data supporting the conclusions of this article will be made available by the authors, without undue reservation.

## Ethics statement

The studies involving human participants were reviewed and approved by The Research Ethics Committee of the Institute of Psychology at the Jagiellonian University. Written informed consent for participation was not required for this study in accordance with the national legislation and the institutional requirements.

## Author contributions

KK, MEM, and JW designed the study. KK, MEM, JW, and MP collected the data. KK, KK-M, AG, and BG performed statistical analyses and/or interpretation. All authors participated in writing a first draft, editing, and contributed to the manuscript, and approved the submitted version.
